# Major Depressive Disorder With Versus Without Psychosocial Triggers: A Secondary Analysis of a Prospective Cohort Study

**DOI:** 10.1155/da/3389394

**Published:** 2026-01-21

**Authors:** Chunfeng Xiao, Shuilin Wu, Lili Shi, Tao Li, Yanping Duan, Xin Yu, Gang Wang, Gang Zhu, Kerang Zhang, Jinya Cao, Jing Wei

**Affiliations:** ^1^ Peking Union Medical College Hospital, Chinese Academy of Medical Sciences and Peking Union Medical College, Beijing, China, cacms.ac.cn; ^2^ Peking University Sixth Hospital (Institute of Mental Health), Beijing, China; ^3^ National Clinical Research Center for Mental Disorders and Key Laboratory for Mental Health, Ministry of Health, Peking University, Beijing, China, pku.edu.cn; ^4^ Beijing Key Laboratory of Mental Disorders, National Clinical Research Center for Mental Disorders and National Center for Mental Disorders, Beijing Anding Hospital, Capital Medical University, Beijing, China, ccmu.edu.cn; ^5^ Advanced Innovation Center for Human Brain Protection, Capital Medical University, Beijing, China, ccmu.edu.cn; ^6^ Department of Psychiatry, The First Affiliated Hospital of China Medical University, Liaoning, China, cmu.edu.cn; ^7^ Department of Psychiatry, First Hospital of Shanxi Medical University, Shanxi Medical University, Taiyuan, Shanxi, China, sxmu.edu.cn

## Abstract

**Background:**

Few studies have examined differences in symptom presentation and antidepressant response between patients with major depressive disorder (MDD) with and without psychosocial triggers.

**Methods:**

This was a secondary analysis of a multicenter, multistage prospective cohort study conducted at nine top tertiary hospitals across six provinces/municipalities in China. The cohort included patients with first‐episode MDD, with or without psychosocial triggers, who received one of six selective serotonin reuptake inhibitors (SSRIs).

**Results:**

Of 359 enrolled patients with first‐episode MDD, 303 (mean [SD] age, 39.6 [10.1] years; 201 [66.8%] women) were included in the final analysis. There were no significant differences in network structure (*M* = 0.40; *p* = 0.97) or global strength (global strength difference [GS] = 0.483; *p* = 0.91) between the two groups. However, network analyses identified distinct core and influential bridge symptoms: psychic anxiety (node strength [Str] = 2.161; bridge strength [BStr] = 1.908) and somatic anxiety (Str = 2.142; BStr = 1.664) in the MDD with psychosocial triggers group, while depressed mood (Str = 3.114; BStr = 2.793) and genital symptoms (Str = 3.085; BStr = 3.085) in the group without psychosocial triggers. There were no significant differences in response rates at all visits. Median time to first response was 4.0 weeks in both groups (log‐rank *p* = 0.23).

**Conclusions:**

While patients with MDD with and without psychosocial triggers shared broadly similar clinical profiles and SSRI responses, differences in symptom network architecture may have implications for individualized symptom monitoring and treatment strategies.

## 1. Introduction

Major depressive disorder (MDD) is a chronic and heterogeneous mental disorder and a leading cause of disability worldwide [1, 2]. Given its varied clinical presentations and complex etiologies, MDD frequently challenges clinicians aiming to provide optimal, individualized treatment. One approach to managing this complexity involves identifying subtypes of MDD characterized by greater homogeneity in clinical features or underlying etiology. Several frameworks have been proposed for subtyping MDD to better capture its clinical diversity. One prominent method focuses on clinical phenotype. For example, the Diagnostic and Statistical Manual of Mental Disorders (Fifth Edition; DSM‐5) introduced specifiers, severity ratings, and cross‐cutting symptom assessments, enabling clinicians to more precisely characterize individual clinical presentations [3]. Within the chapter of depressive disorders, these specifiers include “with mixed features,” “with anxious distress,” among others [4]. Another approach classifies MDD based on treatment response or longitudinal course. A well‐established example is treatment‐resistant depression (TRD), typically defined by insufficient clinical improvement after two or more antidepressant treatment trials [5].

Traditional conceptualizations of MDD proposed that the presence or absence of psychosocial stressors prior to depressive onset defines two etiologically distinct subtypes, historically termed “exogenous” (or reactive) and “endogenous” depression, with differing implications for treatment [6–8]. This distinction has deep roots in psychiatric history and remains conceptually influential in understanding MDD heterogeneity, though no longer included in current diagnostic systems [7]. In this framework, exogenous depression refers to depressive episodes clearly precipitated by external psychosocial events or stressors, such as the death of a loved one, divorce or interpersonal conflicts, job loss, financial hardship, or other major life transitions. In contrast, endogenous depression refers to episodes that arise in the absence of any obvious external trigger. From a clinical symptomatology perspective, endogenous depression has been associated with more pronounced psychomotor disturbances and autonomic symptoms, whereas exogenous depression frequently co‐occurs with anxiety, suggesting potentially distinct underlying physiological mechanisms. A molecular profiling study comparing gene expression patterns in animal models of exogenous versus endogenous depression, as well as in patients with depression, found largely distinct gene expression signatures in the hippocampus for the two subtypes [9]. Furthermore, within exogenous depression, the timing of stress exposure mattered: early‐life (“distal”) versus recent (“proximal”) stressors elicited different molecular profiles, despite some overlapping pathways.

It remains unclear how the presence or absence of psychosocial stressors influences treatment response in patients with MDD. Nevertheless, existing evidence suggests that the two subtypes may involve distinct underlying biological mechanisms that respond differently to treatments [6]. Early studies indicated that patients with endogenous MDD responded more favorably to tricyclic antidepressants (TCAs) compared to selective serotonin reuptake inhibitors (SSRIs) [10]. In a large international trial (iSPOT‐D) evaluating SSRIs and a serotonin‐norepinephrine reuptake inhibitor (SNRI), greater exposure to childhood trauma was associated with lower rates of remission following 8 weeks of antidepressant therapy [11]. Additionally, an individual‐patient meta‐analysis of six randomized clinical trials (RCTs; total *n* = 2858) found significantly poorer outcomes at 3–4 months in patients who experienced severe stressful life events before or during treatment compared with those who did not [12]. However, these findings have not been consistently replicated. Some studies suggest that the influence of life stress on acute antidepressant response may depend on specific contextual factors and the pharmacological mechanism of the medication. For instance, an analysis of the GENDEP trial reported that patients who experienced recent stressful life events exhibited a greater likelihood of response to the SSRI escitalopram over a 12‐week period compared to those without recent stressors; however, no such effect was observed with the TCA nortriptyline [13]. Such discrepancies may result from methodological variations across studies, including substantial heterogeneity in study populations (e.g., differences in first‐episode vs. recurrent depression or unipolar vs. bipolar depression) and inconsistencies in study designs (e.g., interventional trials vs. prospective observational cohort studies).

In 2016, a multicenter, multistage prospective cohort study was initiated in China to identify clinically relevant subtypes of MDD, develop multidimensional diagnostic assessments, and establish combined predictive models aimed at improving diagnostic accuracy and facilitating personalized treatment interventions in patients with MDD [14]. In this cohort, particular attention was directed toward patient‐reported psychosocial stressors closely associated with the onset of depressive episodes. The present secondary analysis aims to compare the clinical symptom networks and antidepressant treatment responses between patients with MDD with and without psychosocial triggers.

## 2. Methods

### 2.1. Design

This study was a secondary analysis based on a multicenter, multistage prospective study conducted across nine clinical sites at leading tertiary hospitals in China (seven academic hospitals and two nonacademic clinical centers), along with one clinical research organization, across six provinces or municipalities. The detailed study protocol and methods have been described previously by Lv et al. [14] and are available at ClinicalTrials.gov (identifier: NCT02023567; registration date: December 2013). Stage 3 of Lv et al. [14], from which data for the present analysis were derived, was a 96‐week longitudinal follow‐up study. It included an 8‐week acute treatment phase and an 88‐week continuation phase, designed to evaluate short‐ and long‐term predictors of treatment response and prognosis. A total of 600 patients with MDD from Stage 1 were treated with one of six SSRIs at standard therapeutic doses for 8 weeks (fluoxetine hydrochloride, 20–60 mg/d; paroxetine hydrochloride, 20–60 mg/d; sertraline hydrochloride, 50–200 mg/d; citalopram, 20–60 mg/d; escitalopram, 10–20 mg/d; or fluvoxamine, 50–300 mg/d). Clinical assessments were conducted at baseline and weeks 2, 4, and 8 during the acute phase and at weeks 24, 48, and 96 during follow‐up.

### 2.2. Participants

Key inclusion criteria were as follows: (1) diagnosis of MDD based on the Chinese version of the Mini‐International Neuropsychiatric Interview (MINI) in accordance with DSM‐IV‐TR criteria, (2) age between 18 and 55 years, (3) a total score of ≥14 on the 17‐item Hamilton Depression Rating Scale (HAMD‐17) at screening, and (4) not receiving regular antidepressant treatment in the past 2 weeks or requiring a change in antidepressant treatment as determined by the treating psychiatrist. Key exclusion criteria included (1) a current or lifetime diagnosis of another psychotic disorder, substance or alcohol dependence, or cognitive impairment; (2) the presence of serious medical conditions, including severe cardiovascular or cerebrovascular disease, respiratory disease, hepatic or renal impairment, or malignancy; and (3) treatment with electroconvulsive therapy (ECT) within the past 3 months. For this secondary analysis, only patients with first‐episode MDD were included to minimize potential confounding effects of prior depressive episodes and to more precisely evaluate the presence or absence of psychosocial stressors preceding illness onset.

### 2.3. Assessment

Assessment of psychosocial triggers was conducted using a predefined simple questionnaire administered by trained psychiatrists who had undergone central training to ensure consistency and reliability among assessors at baseline. Each first‐episode patient with MDD was asked: “Prior to the onset of your symptoms, did you experience any major stressors—such as relationship conflicts, unemployment, financial difficulties, bereavement, or other events that significantly impacted your life?” For ensuring temporal linkage to symptom onset, patients could provide detailed descriptions of the nature and occurrence time about stressor, and finally, responses were recorded in binary form (yes/no), and stressors were briefly documented.

Efficacy was assessed by the HAMD‐17 (range, 0–52; higher scores indicating more severe depressive symptoms) at baseline and 2, 4, 8, 24, 48, and 96 weeks after initiating SSRIs. Response was defined as a 50% reduction in the HAMD‐17. HAMD‐17 includes four factors: (1) somatic anxiety/somatization (items 4 [early insomnia], 5 [middle insomnia], 6 [late insomnia], 11 [somatic anxiety], 13 [general somatic symptoms], and 15 [hypochondria]), (2) psychic anxiety (items 2 [guilt], 9 [agitation], 10 [psychic anxiety], and 17 [insight]), (3) pure depression (items 1 [depressed mood], 7 [work and interests], and 8 [retardation]), and (4) anorexia (items 12 [gastrointestinal symptoms] and 16 [weight loss]) [15]. Items 3 (suicide) and 14 (genital symptoms) were not included in any factors and were treated independently in network analyses.

### 2.4. Statistics

We constructed two symptom networks for MDD patients with and without psychosocial triggers. Specifically, we followed the recommendations by Fried et al. for network analysis: (a) network estimation, (b) network characterization, (c) network stability, and (d) network comparison [16]. Symptom networks were constructed using the qgraph and bootnet packages. Each node in the network represented one of the 17 items from the HAMD‐17 scale, while edges indicated correlations between symptom pairs, and edge weights represent the strength of associations between symptoms. Spearman correlation matrices were used due to the ordinal nature of the data to estimate a nonregularized association. Symptoms were grouped into predefined clinical dimensions based on prior literature: pure depression, psychic anxiety, somatic anxiety/somatization, anorexia, suicide, and genital symptoms. Mean absolute edge weight was computed to assess overall symptom connectivity strength. We calculated the 95% bootstrap interval and the following three common centrality metrics to determine the importance of each symptom within the network: (1) node strength (the sum of the absolute edge weights of edges per node), (2) node betweenness (calculation of the shortest path), and (3) node closeness (the distance between the node and all other nodes by averaging the shortest path lengths to all other nodes) [17]. We estimate the bridge centrality of the nodes within the two symptom clusters. The following three bridge centrality measures were included: (1) bridge strength (BStr) (a total of the absolute values of edges), (2) bridge closeness (average distance from a node to nodes in other communities), and (3) bridge expected influence‐2 step (reflects the indirect impact of the node through other nodes) [18]. Network accuracy was evaluated through nonparametric bootstrapping (1000 iterations), and centrality stability was quantified using the correlation stability (CS) coefficients, with values ≥0.25 indicating moderate stability and ≥0.50 indicating strong stability. Finally, two networks were compared using the network comparison test (NCT) library with 1000 permutations. For the survival analysis of antidepressant treatment response, only patients who received at least one SSRI dose and completed baseline assessments were included.

Missing data were not imputed due to missing completely at random likely (see Table S1 for detailed comparison) and the constraints of this secondary analysis. Multiple imputation with 10 imputations [19] for ensuring the stability of the network results was used for calculating average CS coefficients and the frequency of the core symptoms (the top three in terms of “strength”) appearing in each imputation set.

First time‐to‐response was defined using the HAMD‐17 response criterion (≥50% reduction). A Kaplan–Meier survival model was fit using the survival package, and group differences were assessed with the log‐rank test. Response rates at all visits (weeks 2, 4, 8, 24, 48, and 96) were compared using *χ* ^2^ tests. To ensure the robustness of the results, we additionally employed the Cox proportional hazards model and generalized linear mixed model (GLMM) (analyses used survival and lme4 packages of R software) adjusted for key covariates (age, sex, alcohol category, and baseline HAMD‐17 or HAMA) to explore the association between triggers and treatment responses as sensitivity analyses. All statistical tests were two‐sided, and a *p* value <0.05 was considered statistically significant. All the analyses were conducted in R version 4.4.2.

## 3. Results

### 3.1. Participants

Between November 2013 and January 2016, 359 patients with first‐episode MDD were enrolled. Of these, 303 patients (mean [SD] age, 39.59 [10.09] years; 201 [66.8%] women) reported the psychosocial stressors status prior to illness onset and were included in the network analysis (Table 1, Figure S1).

**Table 1:** Participant demographic and clinical characteristics.

Characteristics ^∗^	Without triggers (*n* = 69)	With triggers (*n* = 234)	*t*/*χ* ^2^	*p*
Sex				
Female	43 (63.2)	158 (67.8)	0.497	0.481
Male	25 (36.8)	75 (32.2)		
First‐class family history				
No	58 (84.1)	195 (83.7)	0.005	0.942
Yes	11 (15.9)	38 (16.3)		
Second‐class family history				
No	62 (89.9)	206 (88.8)	0.061	0.804
Yes	7 (10.1)	26 (11.2)		
Employment				
Full‐time job	50 (72.5)	154 (66.7)	3.011	0.556
Part‐time job	1 (1.4)	6 (2.6)		
Stay home after retirement	6 (8.7)	15 (6.5)		
Awaiting job at home	7 (10.1)	23 (10.0)		
Housewife	5 (7.2)	33 (14.3)		
Occupational essence				
Brain	33 (47.8)	138 (60.5)	5.954	0.051
Physical	22 (31.9)	42 (18.4)		
Brain and physical	14 (20.3)	48 (21.1)		
Marital status				
Married	46 (66.7)	159 (68.8)	1.776	0.777
Divorced	1 (1.4)	9 (3.9)		
Widowed	1 (1.4)	3 (1.3)		
Single	21 (30.4)	59 (25.5)		
Remarried	0 (0.0)	1 (0.4)		
Offspring				
No	25 (36.2)	74 (32.0)	0.423	0.515
Yes	44 (63.8)	157 (68.0)		
Religion				
No	63 (91.3)	216 (93.9)	0.579	0.447
Yes	6 (8.7)	14 (6.1)		
Smoking				
No	51 (73.9)	182 (78.8)	0.757	0.685
Quitter	4 (5.8)	10 (4.3)		
Yes	14 (20.3)	39 (16.9)		
Alcohol				
No	46 (66.7)	155 (67.4)	7.595	0.022
Quitter	8 (11.6)	8 (3.5)		
Yes	15 (21.7)	67 (29.1)		
SSRIs				
Sertraline	11 (28.9)	23 (17.3)	5.913	0.315
Fluoxetine	4 (10.5)	12 (9.0)		
Paroxetine	12 (31.6)	38 (28.6)		
Escitalopram	8 (21.1)	51 (38.3)		
Fluvoxamine	2 (5.3)	3 (2.3)		
Citalopram	1 (2.6)	6 (4.5)		
Age, mean (SD), y	37.65 (10.21)	40.13 (10.03)	−1.27	0.206
Height, mean (SD), cm	166.96 (7.03)	165.31 (0.46)	1.71	0.088
Weight, mean (SD), kg	63.86 (11.43)	61.25 (10.61)	1.76	0.080
Educational level, mean (SD), year	12.72 (3.89)	12.52 (4.12)	0.33	0.741
Baseline HAMD‐17 score, mean (SD)	21.29 (4.56)	21.25 (4.60)	0.48	0.962
Baseline HAMA score, mean (SD)	20.00 (7.97)	18.79 (7.22)	0.18	0.359

^∗^Continuous variables were presented using mean (standard deviation), while categorical variables were presented by frequency (percentage).

### 3.2. Visualized Network for MDD With Psychosocial Triggers

The visualized symptom network for MDD with psychosocial triggers (*n* = 234) is presented in Figure 1A. Edge weights ranged from −0.306 (general somatic symptoms to insight) to 0.416 (psychic anxiety to somatic anxiety). The network average edge weight was 0.059. The strongest associations within this network were observed between psychic anxiety and somatic anxiety (*r* = 0.416), gastrointestinal symptoms and weight loss (*r* = 0.372), and middle insomnia and late insomnia (*r* = 0.362). We estimated the centrality using the centrality metrics of node strength (Str), node closeness (Clo), and node betweenness (Bet). Late insomnia (Str = 2.309, Bet = 24, and Clo = 0.0086), psychic anxiety (Str = 2.161, Bet = 20, and Clo = 0.0085), and somatic anxiety (Str = 2.142, Bet = 22, and Clo = 0.0085) were core symptoms within the network of MDD with psychosocial triggers (Figure 2A, Table 2, and Table S2). Bridge node analysis using BStr, bridge closeness, and bridge expected influence (2‐step) revealed psychic anxiety (BStr = 1.908), insight (BStr = 1.827), gastrointestinal symptoms (BStr = 1.717), and somatic anxiety (BStr = 1.664) as the most influential bridge nodes, suggesting that these symptoms played a critical role in facilitating interactions across different symptom clusters of MDD (Figure 2B).

Figure 1Network analysis visualization for MDD patients with and without psychosocial triggers. Negative edges are shown in red. (A) Network analysis visualization for MDD patients with psychosocial triggers. (B) Network analysis visualization for MDD patients without psychosocial triggers.

**Figure:**
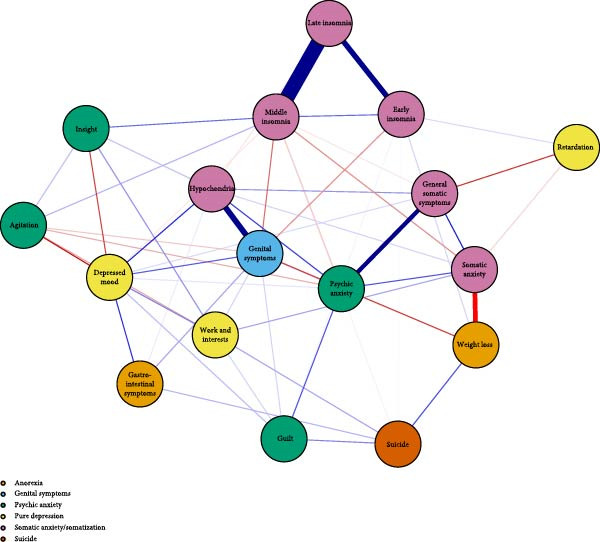
(A)

**Figure:**
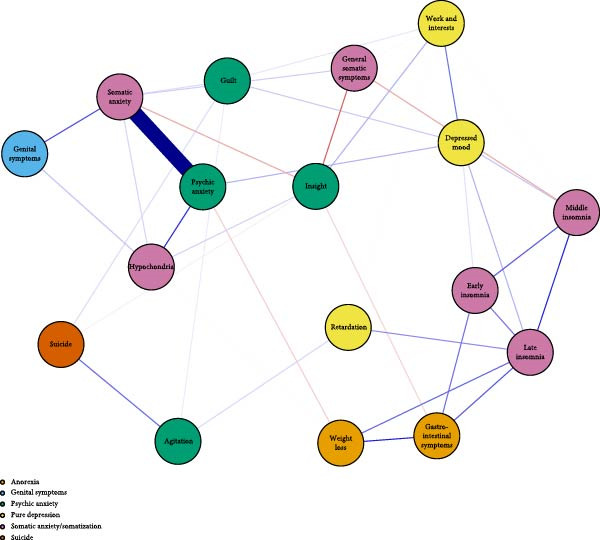
(B)

Figure 2Centrality and bridge centrality indices for MDD patients with psychosocial triggers. (A) Centrality indices for MDD patients with psychosocial triggers. (B) Bridge centrality indices for MDD patients with psychosocial triggers.

**Figure:**
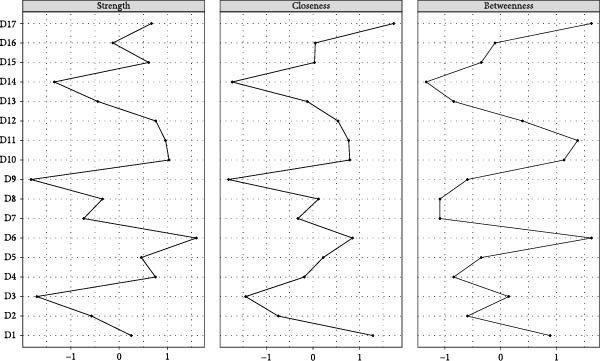
(A)

**Figure:**
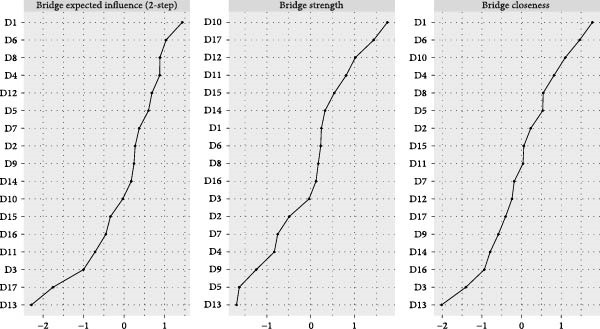
(B)

**Table 2:** Node centrality indices in MDD with and without psychosocial triggers.

Symptoms (item no.)	With psychosocial triggers			Without psychosocial triggers		
	Strength	Closeness	Betweenness	Strength	Closeness	Betweenness
Depressed mood (1)	1.956	0.0089	18	**3.114**	**0.0120**	**32**
Guilty (2)	1.740	0.0074	6	2.119	0.0100	2
Suicide (3)	1.443	0.0068	12	1.797	0.0096	2
Early insomnia (4)	2.088	0.0078	4	2.397	0.0100	6
Middle insomnia (5)	2.010	0.0081	8	**3.204**	**0.0116**	**28**
Late insomnia (6)	**2.309**	**0.0086**	**24**	2.148	0.0097	0
Work and interests (7)	1.698	0.0077	2	2.568	0.0101	6
Retardation (8)	1.800	0.0080	2	1.769	0.0079	0
Agitation (9)	1.411	0.0066	6	2.014	0.0103	0
Psychic anxiety (10)	**2.161**	**0.0085**	**20**	2.858	0.0115	16
Somatic anxiety (11)	**2.142**	**0.0085**	**22**	2.639	0.0117	20
Gastrointestinal symptoms (12)	2.089	0.0083	14	2.307	0.0093	2
General somatic symptoms (13)	1.774	0.0078	4	2.677	0.0011	16
Genital symptoms (14)	1.538	0.0066	0	**3.085**	**0.0129**	**22**
Hypochondria (15)	2.050	0.0080	8	2.989	0.0121	10
Weight loss (16)	1.856	0.0080	10	2.544	0.0107	12
Insight (17)	2.066	0.0093	24	2.162	0.0097	4

*Note:* The bold values indicate the symptoms with the highest node strength scores within their respective groups.

### 3.3. Visualized Network for MDD Without Psychosocial Triggers

The visualized symptom network for MDD without psychosocial triggers (*n* = 69) is presented in Figure 1B. Edge weights ranged from −0.444 (somatic anxiety to weight loss) to 0.574 (middle insomnia to late insomnia). The network average edge weight was 0.056. The strongest symptom associations identified were between middle insomnia and late insomnia (*r* = 0.574), genital symptoms and hypochondriasis (*r* = 0.456), and early insomnia and late insomnia (*r* = 0.452). Centrality metrics identified middle insomnia (Str = 3.204, Bet = 28, and Clo = 0.0116), depressed mood (Str = 3.114, Bet = 32, and Clo = 0.0120), and genital symptoms (Str = 3.085, Bet = 22, and Clo = 0.0129) as the core symptoms within the network of MDD without psychosocial triggers (Figure 3A, Table 2, and Table S3). Bridge node analysis indicated genital symptoms (BStr = 3.085), depressed mood (BStr = 2.793), weight loss (BStr = 2.446), and psychic anxiety (BStr = 2.253) as the most influential bridge nodes, suggesting these symptoms were pivotal in facilitating interactions across different symptom clusters within the MDD without psychosocial triggers network (Figure 3B).

Figure 3Centrality and bridge centrality indices for MDD patients without psychosocial triggers. (A) Centrality indices for MDD patients without psychosocial triggers. (B) Bridge centrality indices for MDD patients without psychosocial triggers.

**Figure:**
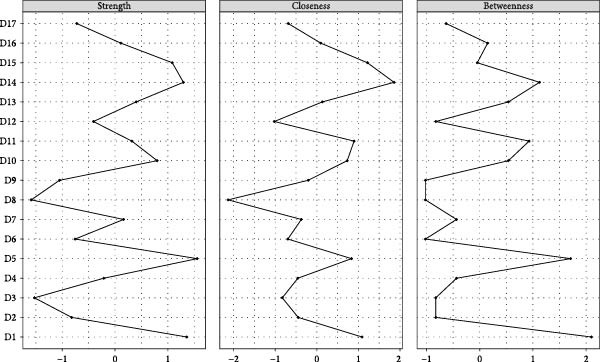
(A)

**Figure:**
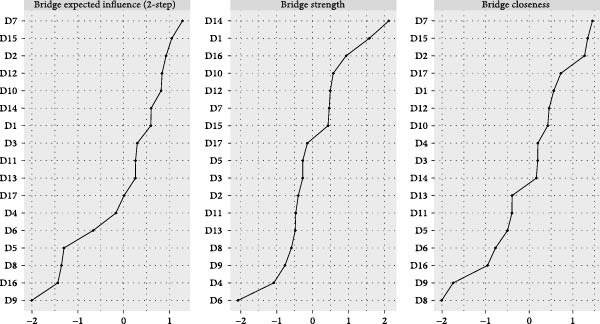
(B)

### 3.4. Network Accuracy and Stability

We assessed the stability of both symptom networks by calculating the CS coefficients for node strength. The CS coefficient was 0.171 for the MDD network with psychosocial triggers and 0.130 for the MDD network without psychosocial triggers (Figures 4A and 5A). The CS coefficients after multiple imputation had significantly improved. The CS coefficient for the MDD network with psychosocial triggers was 0.369, while that in the group without psychosocial triggers was 0.180 (Table S4). The core symptoms that appeared most frequently in the imputation sets were consistent with the primary network analysis results (Table S5). Furthermore, the results of the 95% confidence interval for the edge weights in both networks showed a wide bootstrapped confidence interval around the most estimated edge weights (Figures 4B and 5B).

Figure 4Stability and accuracy of the network for MDD patients with psychosocial triggers. (A) Stability of the network for MDD patients with psychosocial triggers. (B) Accuracy of the network for MDD patients with psychosocial triggers.

**Figure:**
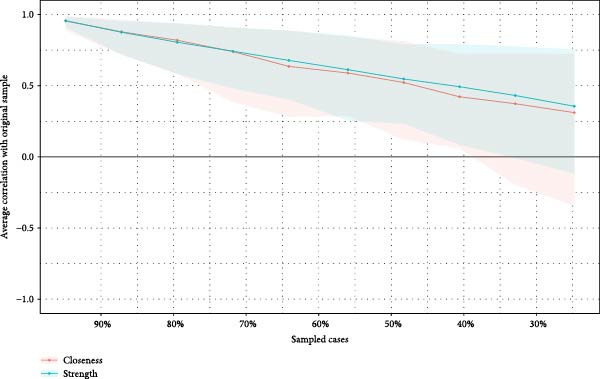
(A)

**Figure:**
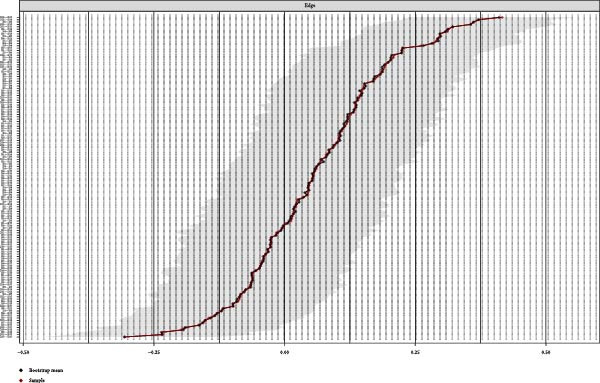
(B)

Figure 5Stability and accuracy of the network for MDD patients without psychosocial triggers. (A) Stability of the network for MDD patients without psychosocial triggers. (B) Accuracy of the network for MDD patients without psychosocial triggers.

**Figure:**
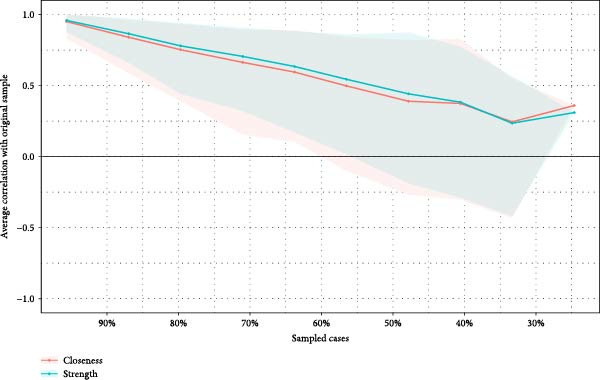
(A)

**Figure:**
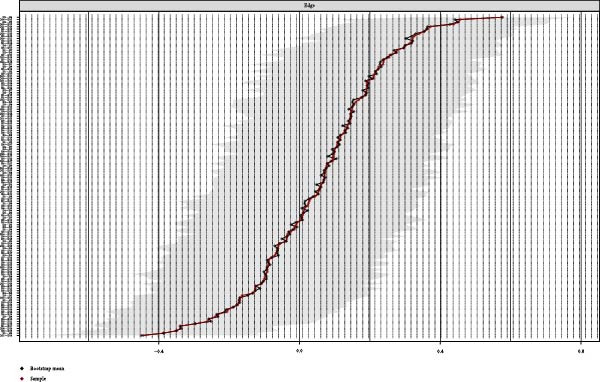
(B)

### 3.5. Network Comparison

We compared the network structure, overall connectivity, and individual edge differences between the two symptom networks. No significant difference was found in the network structure between MDD with psychosocial triggers and MDD without psychosocial triggers (*M* = 0.40; *p* = 0.97). There was also no significant difference in global strength between the two networks (global strength difference [GS] = 0.483; *p* = 0.91).

### 3.6. Comparison of Antidepressant Response Between MDD With and Without Psychosocial Triggers

Patients who completed baseline assessments and received at least one dose of SSRIs according to protocol were included in the final analysis, comprising 133 patients with MDD with psychosocial triggers and 38 patients with MDD without psychosocial triggers (*n* = 171 out of 303; 56.4% retention, Figure S1). Baseline comparisons showed no significant differences between included and excluded groups, supporting the assumption of missing at random (Table S1). There was no significant difference in response rates between the two groups at 2, 4, 8, 24, 48, and 96 weeks (Figure 6A). The median time to first response was 4.0 weeks in both groups (log‐rank *p* = 0.23; Figure 6B). Sensitivity analyses (Cox/GLMM models) further confirmed no SSRI response differences between groups (Tables S6 and S7).

Figure 6The response rates at different visits and Kaplan–Meier curves for response to antidepressants. (A) Bar chart showing response rates (%) across visits (weeks 2–96) for patients with psychosocial triggers (orange) and without triggers (blue). (B) Kaplan–Meier curves showing the proportion of non‐responders over time; the log‐rank test showed no significant difference between groups (*p* = 0.23).

**Figure:**
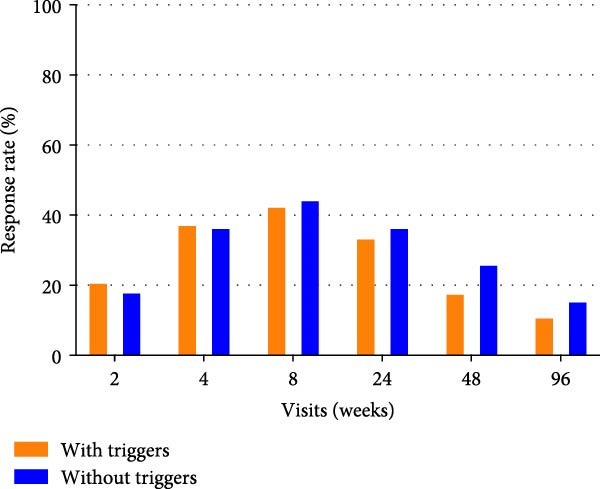
(A)

**Figure:**
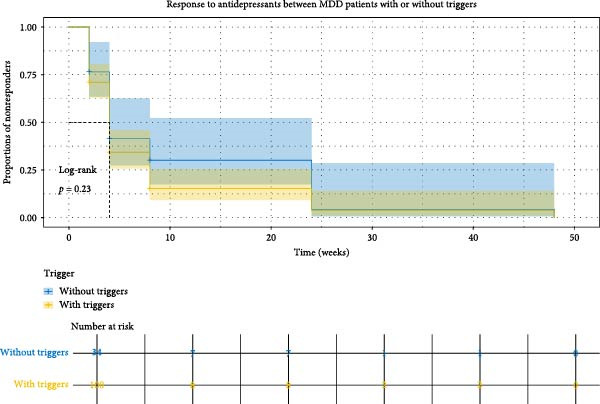
(B)

## 4. Discussion

This secondary analysis of a multicenter, multistage prospective cohort study focused on first‐episode MDD patients who initiated treatment with SSRIs, comparing those with and without psychosocial triggers. The aim was to examine differences in clinical symptom networks and antidepressant treatment response between these two subtypes of MDD. The findings indicated no significant differences between the two groups in most demographic characteristics, clinical features, overall network structure, global connectivity strength, response rates over 96 weeks of SSRI treatment, or time to first treatment response. However, separate network analyses revealed differences in core symptoms and influential bridge nodes. These results suggest that while MDD with and without psychosocial stressors shares a largely similar psychopathological profile, subtle differences in symptom network characteristics may warrant clinical attention.

A substantial body of literature has documented the role of psychosocial triggers in the onset of MDD. However, less is known about how psychosocial triggers preceding the onset of a depressive episode influence clinical characteristics. Most studies have reported that patients with MDD and psychosocial triggers tend to exhibit more severe depressive symptoms and greater functional impairment than those without such triggers. For example, a cross‐sectional study by Muscatell et al. [20] found that patients with MDD who experienced a severe life event prior to illness onset demonstrated higher overall depression severity, reported more cognitive and somatic symptoms, and had lower functioning compared with patients without preonset severe life events. In contrast, our study found no significant differences in depression severity or anxiety severity between the two MDD subtypes, nor in overall network structure. This result is consistent with the findings of a network analysis conducted on a large sample cohort in the UK (the GLAD Study), which indicated that there were no differences in the depression and anxiety symptom networks between those who reported lifetime trauma and those who did not on any metric [21]. Although differences in core symptoms emerged between the two networks, these may reflect subtle variations rather than fundamental reconfiguration. One possible explanation is that, unlike most previous studies, our analysis focused exclusively on patients with first‐episode MDD. By excluding patients with recurrent episodes, we minimized confounding effects related to illness chronicity and neuroprogression, offering a more direct test of whether initial presentation is shaped by trigger context. Overall, these findings suggest that psychosocial stress may subtly influence specific symptom patterns without altering overall severity or network structure, highlighting the importance of network‐based approaches in characterizing MDD subtypes.

It is well recognized that in current diagnostic practice, endogenous depression is no longer an official diagnostic label, but its clinical features are acknowledged through specifiers. The DSM‐5 specifier “with melancholic features” essentially reflects the modern counterpart of the traditional endogenous concept, characterized by persistently low and nonreactive mood, anhedonia, disturbed sleep and appetite, and weight loss [22, 23]. In our study, depressed mood and general somatic symptoms emerged as core symptoms and influential bridge nodes within the network of MDD without psychosocial triggers. These findings are consistent with established understanding of this MDD subtype and underscore the central role of depressed mood and general physical complaints as highly identifiable and pivotal symptoms, suggesting that targeting these symptoms in treatment may help reduce overall depressive symptom severity and disrupt pathways between symptom clusters. Ghaemi and Vohringer have emphasized that most depressive conditions are influenced equally by genetic and environmental factors and noted that neurotic (or reactive) depression presents a markedly different psychopathological profile compared with melancholia [24, 25]. Prior research has shown that neurotic or reactive depression is closely associated with anxiety, mood reactivity, and heightened sensitivity to psychosocial stressors and has raised questions about the potential merits and risks of benzodiazepine use in treating patients with reactive depression. In line with these findings, our study identified psychic anxiety and somatic anxiety as core symptoms and influential bridge nodes in the network of MDD with psychosocial triggers. This suggests that both overall symptom reduction and disruption of interactions across symptom clusters in this group may require particular attention to anxiety symptoms. This effect of anxiety expression may not entirely stem from the classification of psychosocial triggers, as stress can also strongly correlate with depressive symptoms. In a network analysis of negative life events and depression or anxiety, stress showed a strong correlation with depressive symptoms such as sadness, beyond anxiety alone [26]. These findings highlight the clinical importance of distinguishing between MDD with and without psychosocial triggers and tailoring treatment approaches accordingly. For example, in patients with MDD with psychosocial triggers, careful assessment and management of anxiety symptoms—including both psychic and somatic components—may be especially critical, and the use of anxiolytic agents could be considered when appropriate.

MDD with and without psychosocial triggers may also differ substantially in treatment response. Melancholic depression has been reported to show greater responsiveness to certain antidepressants compared with neurotic (reactive) depression [24]. Furthermore, the presence of melancholic features has been associated with poorer response to psychotherapy and placebo but relatively better response to antidepressants and ECT [27]. However, in our study, no significant differences were observed between the two subtypes in response to SSRIs, including both response rates and time to response. We recognize that several factors may account for this inconsistency. In addition to our focus on SSRIs as a single class of treatment, our study population was restricted to patients with first‐episode MDD, which differs from many prior studies. Another potential factor is variation in how psychosocial triggers are defined; in our study, assessment was based on patient self‐report of psychosocial stressors they perceived as closely related to illness onset, aiming to capture subjective experience that may not be fully reflected by structured self‐assessment tools. A cross‐sectional study in Japan reported that 85% of psychiatrists considered antidepressants appropriate for MDD patients with melancholic features, compared with only 40% for those with reactive features [25]. Our findings suggest that psychiatrists should adopt a more proactive stance toward prescribing SSRIs for patients with MDD with psychosocial triggers, like their approach for patients without such triggers.

Some studies comparing melancholic and nonmelancholic depression have suggested that melancholia may be associated with more pronounced biological abnormalities, such as elevated baseline cortisol levels and impaired dexamethasone suppression, whereas atypical (often stress‐linked) depression may present with lower morning cortisol levels but elevated inflammatory markers [28, 29]. However, much of the available evidence has not been replicated, studies have focused on different biomarkers, and many reports are based on limited sample sizes. Despite these clues, it is important to emphasize that there is extensive overlap, especially monoamine dysregulations. Contemporary psychiatry increasingly adopts a biopsychosocial framework, recognizing that each depressive episode arises from the interaction of brain physiology, genetic predisposition, and environmental stress. We acknowledge that these biological differences and overlaps may partly account for the variations observed in symptom network characteristics while also explaining the shared core psychopathological features.

## 5. Limitations

This analysis has several limitations. First, our study population was restricted to patients with first‐episode MDD, which strengthens internal validity but may limit the applicability of results to patients with recurrent MDD, where psychosocial stressors and biological underpinnings may differ. Similarly, the external validity is also limited as we only included the Chinese population. Therefore, replication in more diverse international samples is warranted to determine the broader generalizability of these findings. Second, the assessment of psychosocial triggers relied on patient self‐report, which, although designed to capture the subjective experience of stressors linked to illness onset, may introduce recall bias or subjective interpretation that could affect classification. However, our data collectors were professional clinicians, and the baseline diagnosis and stressor collection were conducted simultaneously, with detailed inquiries into the nature and timing of the patient’s stressors, thereby reducing recall and measurement biases to some extent. Nonetheless, future studies are recommended to use standardized structured questionnaires to further minimize measurement bias. Third, the network analyses, although informative, are inherently exploratory and observational. The limited stability coefficients and relatively wide confidence intervals around edge weight estimates indicate that findings related to network structure, including core and bridge symptoms, should be interpreted with caution as exploratory results, particularly given the smaller sample size in the MDD without psychosocial triggers group. The multiple imputation analysis also highlighted the significance of the sample size and further enhanced the exploratory nature of the study. Future confirmatory studies should ensure adequate sample size. Fourth, the antidepressant response analysis was limited by substantial missing data due to incomplete follow‐up assessments. This attrition may introduce selection bias, as patients who remained likely had better treatment adherence or less severe symptoms, potentially limiting the generalizability of antidepressant response findings. Although the assumption of missing completely at random reduces bias within the potential range, future studies should implement enhanced follow‐up management strategies, such as more frequent patient contact or incentives for participation, to minimize attrition and improve data completeness. Finally, by aggregating all psychosocial triggers into a single category, we assumed homogeneity in their effects on symptom networks and treatment response. This approach may overlook potential variations based on trigger type, combination, or load. Future studies with larger cohorts may conduct exploratory analyses to examine subtype‐specific effects.

## 6. Conclusion

This study provides preliminary evidence highlighting differences in clinical symptom characteristics and response to SSRIs between MDD with and without psychosocial triggers within the same prospective cohort. Understanding differences in symptom network architecture between MDD with and without psychological triggers could help optimize antidepressant strategies, particularly by targeting anxiety symptoms in psychosocially triggered cases and addressing depressed mood more directly in patients without such triggers.

## Ethics Statement

All participants provided written consent prior to enrollment. This study had been approved by the Ethic Committee of Peking University Sixth Hospital (Approval o. 2013‐29‐1), and the trial was registered with ClinicalTrials.gov in December 2013.

## Disclosure

The funders had no role in the design and conduct of the study; collection, management, analysis, and interpretation of the data; preparation, review, or approval of the manuscript; and decision to submit the manuscript for publication. Dr. Jing Wei and Dr. Jinya Cao had full access to all data in the study and took responsibility for the integrity of the data and the accuracy of the data analysis.

## Conflicts of Interest

The authors declare no conflicts of interest.

## Author Contributions

Concept and design: **Chunfeng Xiao**, **Jinya Cao**, and **Jing Wei**. Acquisition, analysis, or interpretation of data: **Chunfeng Xiao**, **Lili Shi**, **Tao Li**, and **Yanping Duan**. Drafting of the manuscript: **Chunfeng Xiao** and **Shuilin Wu**. Critical review of the manuscript for important intellectual content: **Jinya Cao** and **Jing Wei**. Statistical analysis: **Chunfeng Xiao** and **Shuilin Wu**. Obtained funding: **Jinya Cao** and **Jing Wei**. Administrative, technical, or material support: **Jinya Cao**, **Jing Wei**, **Xin Yu**, **Gang Wang**, **Gang Zhu**, and **Kerang Zhang**. Supervision: **Jinya Cao** and **Jing Wei**. Chunfeng Xiao and Shuilin Wu contributed equally to this work.

## Funding

This work was supported by research grants from the National Key Basic Research Program of China (No. 2013CB531305) and the STI2030‐Major Project (2021ZD0202001).

## Supporting Information

Additional supporting information can be found online in the Supporting Information section.

## Supporting information


**Supporting Information** Table S1: It presents the comparison of demographic characteristics between included and excluded participants. Table S2: It presents the edge weights with bootstrapped 95% CI for MDD patients with psychosocial triggers. Table S3: It presents the edge weights with bootstrapped 95% CI for MDD patients without psychosocial triggers. Table S4: It presents the average correlation stability coefficients of multiple imputation. Table S5: It presents the frequency of core symptoms appearing in imputation sets. Table S6: It presents the association analysis of Cox proportional hazards model between psychosocial triggers and treatment response. Table S7: It presents the association analysis of the GLMM model between psychosocial triggers and treatment response at different time points. Figure S1: It presents the flowchart for the inclusion and exclusion of the population.

## Data Availability

The data that support the findings of this study are available from the corresponding author upon reasonable request.
