# Supplementary material for: Major Depressive Disorder With Versus Without Psychosocial Triggers: A Secondary Analysis of a Prospective Cohort Study
Source: Depress Anxiety. 2026 Jan 21;2026:3389394. doi: 10.1155/da/3389394 (PMC12820511; doi:10.1155/da/3389394)
Supplement: Supplementary file 1 — Supporting Information Table S1: It presents the comparison of demographic characteristics between included and excluded participants. Table S2: It presents the edge weights with bootstrapped 95% CI for MDD patients with psychosocial triggers. Table S3: It presents the edge weights with bootstrapped 95% CI for MDD patients without psychosocial triggers. Table S4: It presents the average correlation stability coefficients of multiple imputation. Table S5: It presents the frequency of core symptoms appearing in imputation sets. Table S6: It presents the association analysis of Cox proportional hazards model between psychosocial triggers and treatment response. Table S7: It presents the association analysis of the GLMM model between psychosocial triggers and treatment response at different time points. Figure S1: It presents the flowchart for the inclusion and exclusion of the population. [file DA-2026-3389394-s001.doc]

**Table S1.** Demographic characteristics comparison of included and excluded participants.

| Characteristics^*^ | Included (n=171) | Excluded (n=188) | *P* |
| --- | --- | --- | --- |
| Age, mean (SD), y | 40.24 (10.13) | 41.04 (10.31) | 0.651 |
| Height, mean (SD), cm | 166.13 (7.36) | 165.20 (6.79) | 0.217 |
| Weight, mean (SD), kg | 61.79 (10.74) | 61.64 (11.08) | 0.902 |
| Sex |  |  | 0.196 |
| Male | 48 (28.1) | 64 (34.4) |  |
| Female | 123 (71.9) | 122 (65.6) |  |
| Education level |  |  | 0.292 |
| Illiteracy | 0 (0.0) | 1 (0.5) |  |
| Primary school | 3 (1.8) | 11 (5.9) |  |
| Junior high school | 42 (25.0) | 50 (26.6) |  |
| High school | 31 (18.5) | 39 (20.7) |  |
| College | 79 (47.0) | 79 (42.0) |  |
| Master's degree or above | 13 (7.7) | 8 (4.3) |  |
| First-class family history |  |  | 1.000 |
| No | 144 (84.2) | 157 (84.0) |  |
| Yes | 27 (15.8) | 30 (16.0) |  |
| Second-class family history |  |  | 0.246 |
| No | 148 (86.5) | 169 (90.9) |  |
| Yes | 23 (13.5) | 17 (9.1) |  |
| Employment |  |  | 0.150 |
| Full-time job | 119 (70.8) | 126 (67.0) |  |
| Part-time job | 6 (3.6) | 1 (0.5) |  |
| Stay home after retirement | 8 (4.8) | 16 (8.5) |  |
| Awaiting job at home | 16 (9.5) | 19 (10.1) |  |
| Housewife | 19 (11.3) | 26 (13.8) |  |
| Marital status |  |  | 0.171 |
| Married | 112 (66.7) | 130 (69.1) |  |
| Divorced | 7 (4.2) | 6 (3.2) |  |
| Widowed | 3 (1.8) | 1 (0.5) |  |
| Single | 45 (26.8) | 51 (27.1) |  |
| Remarried | 1 (0.6) | 0 (0.0) |  |
| Offspring |  |  | 0.434 |
| No | 59 (35.1) | 58 (30.9) |  |
| Yes | 109 (64.9) | 130 (69.1) |  |
| Religion |  |  | 0.553 |
| No | 153 (91.1) | 174 (93.0) |  |
| Yes | 15 (8.9) | 13 (7.0) |  |
| Smoking |  |  | 0.326 |
| No | 126 (75.0) | 153 (81.4) |  |
| Quitter | 9 (5.4) | 7 (3.7) |  |
| Yes | 33 (19.6) | 28 (14.9) |  |
| Alcohol |  |  | 0.113 |
| No | 105 (62.5) | 136 (72.7) |  |
| Quitter | 11 (6.5) | 10 (5.3) |  |
| Yes | 52 (31.0) | 41 (21.9) |  |

^*^Continuous variables were presented using mean (standard deviation), while categorical variables were presented by frequency (percentage).

**Table S2.** Edge weights with bootstrapped 95% CI for MDD patients with psychosocial triggers.

| Node Pair | Edge Weight | 95% CI Lower | 95% CI Upper |
| --- | --- | --- | --- |
| depressed mood-guilt | 0.202 | 0.033 | 0.356 |
| depressed mood-suicide | 0.022 | -0.171 | 0.216 |
| depressed mood-early insomnia | 0.178 | 0.020 | 0.336 |
| depressed mood-middle insomnia | 0.208 | 0.047 | 0.369 |
| depressed mood-late insomnia | 0.225 | 0.067 | 0.380 |
| depressed mood-work and interests | 0.290 | 0.125 | 0.462 |
| depressed mood-retardation | 0.150 | -0.026 | 0.318 |
| depressed mood-agitation | -0.003 | -0.172 | 0.145 |
| depressed mood-psychic anxiety | 0.225 | 0.049 | 0.398 |
| depressed mood-somatic anxiety | 0.119 | -0.062 | 0.296 |
| depressed mood-gastrointestinal symptoms | 0.055 | -0.119 | 0.224 |
| depressed mood-general somatic symptoms | -0.032 | -0.200 | 0.133 |
| depressed mood-genital symptoms | -0.061 | -0.236 | 0.114 |
| depressed mood-hypochondria | 0.104 | -0.066 | 0.282 |
| depressed mood-weight loss | 0.079 | -0.091 | 0.244 |
| depressed mood-insight | 0.002 | -0.164 | 0.160 |
| guilt-suicide | 0.184 | -0.008 | 0.358 |
| guilt-early insomnia | 0.044 | -0.134 | 0.225 |
| guilt-middle insomnia | 0.061 | -0.118 | 0.235 |
| guilt-late insomnia | -0.121 | -0.281 | 0.044 |
| guilt-work and interests | 0.173 | 0.003 | 0.336 |
| guilt-retardation | 0.045 | -0.127 | 0.205 |
| guilt-agitation | 0.168 | -0.023 | 0.346 |
| guilt-psychic anxiety | 0.135 | -0.035 | 0.306 |
| guilt-somatic anxiety | 0.185 | 0.004 | 0.366 |
| guilt-gastrointestinal symptoms | 0.048 | -0.137 | 0.214 |
| guilt-general somatic symptoms | -0.039 | -0.193 | 0.120 |
| guilt-genital symptoms | 0.121 | -0.060 | 0.303 |
| guilt-hypochondria | 0.069 | -0.095 | 0.239 |
| guilt-weight loss | -0.032 | -0.199 | 0.151 |
| guilt-insight | 0.111 | -0.058 | 0.261 |
| suicide-early insomnia | 0.065 | -0.107 | 0.227 |
| suicide-middle insomnia | 0.012 | -0.170 | 0.181 |
| suicide-late insomnia | 0.044 | -0.129 | 0.227 |
| suicide-work and interests | -0.084 | -0.253 | 0.090 |
| suicide-retardation | 0.046 | -0.109 | 0.221 |
| suicide-agitation | 0.297 | 0.123 | 0.454 |
| suicide-psychic anxiety | -0.044 | -0.223 | 0.137 |
| suicide-somatic anxiety | -0.134 | -0.299 | 0.028 |
| suicide-gastrointestinal symptoms | 0.110 | -0.066 | 0.276 |
| suicide-general somatic symptoms | -0.016 | -0.183 | 0.140 |
| suicide-genital symptoms | -0.062 | -0.233 | 0.098 |
| suicide-hypochondria | -0.145 | -0.316 | 0.024 |
| suicide-weight loss | 0.014 | -0.152 | 0.173 |
| suicide-insight | -0.165 | -0.317 | -0.006 |
| early insomnia-middle insomnia | 0.312 | 0.143 | 0.460 |
| early insomnia-late insomnia | 0.284 | 0.113 | 0.442 |
| early insomnia-work and interests | 0.061 | -0.095 | 0.237 |
| early insomnia-retardation | 0.105 | -0.050 | 0.262 |
| early insomnia-agitation | 0.121 | -0.051 | 0.280 |
| early insomnia-psychic anxiety | -0.087 | -0.249 | 0.079 |
| early insomnia-somatic anxiety | -0.037 | -0.183 | 0.135 |
| early insomnia-gastrointestinal symptoms | 0.292 | 0.125 | 0.455 |
| early insomnia-general somatic symptoms | -0.130 | -0.304 | 0.029 |
| early insomnia-genital symptoms | 0.104 | -0.072 | 0.276 |
| early insomnia-hypochondria | -0.089 | -0.250 | 0.065 |
| early insomnia-weight loss | 0.122 | -0.049 | 0.285 |
| early insomnia-insight | 0.057 | -0.114 | 0.206 |
| middle insomnia-late insomnia | 0.362 | 0.200 | 0.506 |
| middle insomnia-work and interests | 0.153 | -0.018 | 0.327 |
| middle insomnia-retardation | 0.085 | -0.095 | 0.247 |
| middle insomnia-agitation | -0.068 | -0.248 | 0.110 |
| middle insomnia-psychic anxiety | -0.061 | -0.224 | 0.117 |
| middle insomnia-somatic anxiety | -0.027 | -0.195 | 0.154 |
| middle insomnia-gastrointestinal symptoms | 0.086 | -0.104 | 0.258 |
| middle insomnia-general somatic symptoms | -0.233 | -0.395 | -0.070 |
| middle insomnia-genital symptoms | 0.024 | -0.149 | 0.174 |
| middle insomnia-hypochondria | -0.048 | -0.225 | 0.14 |
| middle insomnia-weight loss | 0.126 | -0.042 | 0.29 |
| middle insomnia-insight | 0.144 | -0.048 | 0.314 |
| late insomnia-work and interests | 0.023 | -0.134 | 0.199 |
| late insomnia-retardation | 0.262 | 0.093 | 0.425 |
| late insomnia-agitation | 0.079 | -0.088 | 0.251 |
| late insomnia-psychic anxiety | -0.046 | -0.200 | 0.108 |
| late insomnia-somatic anxiety | -0.026 | -0.192 | 0.138 |
| late insomnia-gastrointestinal symptoms | 0.314 | 0.167 | 0.456 |
| late insomnia-general somatic symptoms | -0.098 | -0.259 | 0.058 |
| late insomnia-genital symptoms | 0.056 | -0.108 | 0.232 |
| late insomnia-hypochondria | -0.026 | -0.197 | 0.141 |
| late insomnia-weight loss | 0.298 | 0.145 | 0.453 |
| late insomnia-insight | -0.044 | -0.208 | 0.118 |
| work and interests-retardation | 0.152 | -0.030 | 0.323 |
| work and interests-agitation | 0.018 | -0.161 | 0.197 |
| work and interests-psychic anxiety | 0.134 | -0.030 | 0.302 |
| work and interests-somatic anxiety | 0.015 | -0.159 | 0.194 |
| work and interests-gastrointestinal symptoms | 0.016 | -0.170 | 0.166 |
| work and interests-general somatic symptoms | -0.155 | -0.308 | 0.008 |
| work and interests-genital symptoms | 0.047 | -0.122 | 0.217 |
| work and interests-hypochondria | 0.115 | -0.054 | 0.288 |
| work and interests-weight loss | 0.036 | -0.115 | 0.192 |
| work and interests-insight | 0.226 | 0.053 | 0.383 |
| retardation-agitation | 0.189 | 0.023 | 0.361 |
| retardation-psychic anxiety | 0.053 | -0.12 | 0.228 |
| retardation-somatic anxiety | -0.118 | -0.28 | 0.052 |
| retardation-gastrointestinal symptoms | 0.135 | -0.034 | 0.309 |
| retardation-general somatic symptoms | -0.040 | -0.200 | 0.129 |
| retardation-genital symptoms | -0.002 | -0.179 | 0.187 |
| retardation-hypochondria | 0.147 | -0.031 | 0.311 |
| retardation-weight loss | 0.132 | -0.023 | 0.296 |
| retardation-insight | 0.139 | -0.036 | 0.291 |
| agitation-psychic anxiety | 0.052 | -0.121 | 0.210 |
| agitation-somatic anxiety | -0.026 | -0.194 | 0.143 |
| agitation-gastrointestinal symptoms | 0.148 | -0.019 | 0.320 |
| agitation-general somatic symptoms | -0.063 | -0.221 | 0.095 |
| agitation-genital symptoms | -0.007 | -0.192 | 0.177 |
| agitation-hypochondria | 0.018 | -0.150 | 0.172 |
| agitation-weight loss | 0.093 | -0.077 | 0.258 |
| agitation-insight | -0.062 | -0.219 | 0.105 |
| psychic anxiety-somatic anxiety | 0.416 | 0.250 | 0.559 |
| psychic anxiety-gastrointestinal symptoms | -0.054 | -0.232 | 0.109 |
| psychic anxiety-general somatic symptoms | 0.099 | -0.063 | 0.277 |
| psychic anxiety-genital symptoms | 0.136 | -0.006 | 0.303 |
| psychic anxiety-hypochondria | 0.356 | 0.185 | 0.505 |
| psychic anxiety-weight loss | -0.198 | -0.353 | -0.034 |
| psychic anxiety-insight | -0.065 | -0.245 | 0.124 |
| somatic anxiety-gastrointestinal symptoms | -0.016 | -0.199 | 0.159 |
| somatic anxiety-general somatic symptoms | 0.198 | 0.035 | 0.350 |
| somatic anxiety-genital symptoms | 0.322 | 0.154 | 0.483 |
| somatic anxiety-hypochondria | 0.191 | 0.016 | 0.361 |
| somatic anxiety-weight loss | -0.079 | -0.251 | 0.077 |
| somatic anxiety-insight | -0.234 | -0.390 | -0.056 |
| gastrointestinal symptoms-general somatic symptoms | 0.096 | -0.086 | 0.273 |
| gastrointestinal symptoms-genital symptoms | 0.071 | -0.088 | 0.227 |
| gastrointestinal symptoms-hypochondria | -0.085 | -0.274 | 0.095 |
| gastrointestinal symptoms-weight loss | 0.372 | 0.223 | 0.504 |
| gastrointestinal symptoms-insight | -0.191 | -0.364 | -0.007 |
| general somatic symptoms-genital symptoms | 0.143 | -0.022 | 0.305 |
| general somatic symptoms-hypochondria | 0.103 | -0.069 | 0.281 |
| general somatic symptoms-weight loss | 0.022 | -0.144 | 0.199 |
| general somatic symptoms-insight | -0.306 | -0.465 | -0.132 |
| genital symptoms-hypochondria | 0.209 | 0.017 | 0.372 |
| genital symptoms-weight loss | -0.074 | -0.239 | 0.091 |
| genital symptoms-insight | -0.099 | -0.234 | 0.049 |
| hypochondria-weight loss | -0.152 | -0.334 | 0.011 |
| hypochondria-insight | 0.193 | 0.024 | 0.380 |
| weight loss-insight | -0.027 | -0.189 | 0.131 |

**Table S3.** Edge weights with bootstrapped 95% CI for MDD patients without psychosocial triggers.

| Node Pair | Edge Weight | 95% CI Lower | 95% CI Upper |
| --- | --- | --- | --- |
| depressed mood-guilt | 0.216 | -0.113 | 0.53 |
| depressed mood-suicide | 0.108 | -0.205 | 0.401 |
| depressed mood-early insomnia | 0.025 | -0.317 | 0.375 |
| depressed mood-middle insomnia | -0.169 | -0.448 | 0.163 |
| depressed mood-late insomnia | -0.015 | -0.365 | 0.327 |
| depressed mood-work and interests | 0.277 | -0.055 | 0.583 |
| depressed mood-retardation | 0.044 | -0.301 | 0.383 |
| depressed mood-agitation | -0.387 | -0.628 | -0.131 |
| depressed mood-psychic anxiety | 0.175 | -0.104 | 0.453 |
| depressed mood-somatic anxiety | 0.056 | -0.256 | 0.321 |
| depressed mood-gastrointestinal symptoms | 0.354 | 0.090 | 0.586 |
| depressed mood-general somatic symptoms | 0.186 | -0.110 | 0.496 |
| depressed mood-genital symptoms | 0.314 | -0.008 | 0.589 |
| depressed mood-hypochondria | 0.363 | 0.051 | 0.619 |
| depressed mood-weight loss | -0.088 | -0.386 | 0.208 |
| depressed mood-insight | -0.337 | -0.583 | -0.042 |
| guilt-suicide | 0.273 | -0.044 | 0.558 |
| guilt-early insomnia | 0.159 | -0.178 | 0.471 |
| guilt-middle insomnia | 0.061 | -0.231 | 0.368 |
| guilt-late insomnia | -0.010 | -0.322 | 0.319 |
| guilt-work and interests | 0.191 | -0.137 | 0.482 |
| guilt-retardation | 0.072 | -0.229 | 0.385 |
| guilt-agitation | -0.039 | -0.352 | 0.260 |
| guilt-psychic anxiety | 0.320 | 0.063 | 0.554 |
| guilt-somatic anxiety | 0.136 | -0.227 | 0.456 |
| guilt-gastrointestinal symptoms | -0.097 | -0.428 | 0.252 |
| guilt-general somatic symptoms | 0.058 | -0.235 | 0.372 |
| guilt-genital symptoms | 0.192 | -0.142 | 0.498 |
| guilt-hypochondria | 0.15 | -0.189 | 0.459 |
| guilt-weight loss | 0.114 | -0.223 | 0.407 |
| guilt-insight | 0.031 | -0.250 | 0.320 |
| suicide-early insomnia | 0.154 | -0.202 | 0.470 |
| suicide-middle insomnia | -0.163 | -0.498 | 0.169 |
| suicide-late insomnia | -0.061 | -0.395 | 0.252 |
| suicide-work and interests | 0.237 | -0.093 | 0.539 |
| suicide-retardation | -0.070 | -0.360 | 0.246 |
| suicide-agitation | 0.010 | -0.305 | 0.315 |
| suicide-psychic anxiety | -0.026 | -0.328 | 0.274 |
| suicide-somatic anxiety | 0.009 | -0.306 | 0.297 |
| suicide-gastrointestinal symptoms | 0.224 | -0.118 | 0.538 |
| suicide-general somatic symptoms | -0.036 | -0.341 | 0.240 |
| suicide-genital symptoms | -0.006 | -0.365 | 0.361 |
| suicide-hypochondria | 0.028 | -0.312 | 0.352 |
| suicide-weight loss | 0.326 | 0.014 | 0.584 |
| suicide-insight | -0.067 | -0.381 | 0.255 |
| early insomnia-middle insomnia | 0.299 | 0.007 | 0.558 |
| early insomnia-late insomnia | 0.452 | 0.173 | 0.710 |
| early insomnia-work and interests | 0.106 | -0.232 | 0.436 |
| early insomnia-retardation | 0.188 | -0.123 | 0.458 |
| early insomnia-agitation | 0.013 | -0.307 | 0.344 |
| early insomnia-psychic anxiety | 0.014 | -0.307 | 0.348 |
| early insomnia-somatic anxiety | 0.069 | -0.251 | 0.384 |
| early insomnia-gastrointestinal symptoms | 0.147 | -0.174 | 0.490 |
| early insomnia-general somatic symptoms | -0.125 | -0.417 | 0.175 |
| early insomnia-genital symptoms | -0.258 | -0.540 | 0.038 |
| early insomnia-hypochondria | -0.089 | -0.413 | 0.236 |
| early insomnia-weight loss | 0.191 | -0.120 | 0.476 |
| early insomnia-insight | 0.107 | -0.238 | 0.432 |
| middle insomnia-late insomnia | 0.574 | 0.331 | 0.774 |
| middle insomnia-work and interests | 0.007 | -0.300 | 0.325 |
| middle insomnia-retardation | 0.118 | -0.158 | 0.404 |
| middle insomnia-agitation | 0.224 | -0.083 | 0.514 |
| middle insomnia-psychic anxiety | -0.203 | -0.508 | 0.130 |
| middle insomnia-somatic anxiety | -0.261 | -0.537 | 0.061 |
| middle insomnia-gastrointestinal symptoms | -0.125 | -0.454 | 0.203 |
| middle insomnia-general somatic symptoms | -0.173 | -0.463 | 0.114 |
| middle insomnia-genital symptoms | -0.296 | -0.586 | 0.026 |
| middle insomnia-hypochondria | -0.101 | -0.412 | 0.249 |
| middle insomnia-weight loss | 0.134 | -0.176 | 0.449 |
| middle insomnia-insight | 0.296 | -0.040 | 0.566 |
| late insomnia-work and interests | 0.099 | -0.210 | 0.408 |
| late insomnia-retardation | -0.133 | -0.443 | 0.189 |
| late insomnia-agitation | 0.077 | -0.233 | 0.385 |
| late insomnia-psychic anxiety | -0.091 | -0.388 | 0.212 |
| late insomnia-somatic anxiety | 0.052 | -0.275 | 0.356 |
| late insomnia-gastrointestinal symptoms | 0.095 | -0.230 | 0.377 |
| late insomnia-general somatic symptoms | 0.068 | -0.229 | 0.395 |
| late insomnia-genital symptoms | -0.036 | -0.373 | 0.289 |
| late insomnia-hypochondria | -0.176 | -0.461 | 0.149 |
| late insomnia-weight loss | 0.131 | -0.187 | 0.425 |
| late insomnia-insight | 0.080 | -0.245 | 0.371 |
| work and interests-retardation | -0.098 | -0.446 | 0.254 |
| work and interests-agitation | -0.239 | -0.545 | 0.075 |
| work and interests-psychic anxiety | 0.115 | -0.176 | 0.401 |
| work and interests-somatic anxiety | 0.240 | -0.049 | 0.493 |
| work and interests-gastrointestinal symptoms | 0.145 | -0.172 | 0.452 |
| work and interests-general somatic symptoms | 0.141 | -0.169 | 0.420 |
| work and interests-genital symptoms | 0.191 | -0.111 | 0.481 |
| work and interests-hypochondria | 0.143 | -0.158 | 0.428 |
| work and interests-weight loss | -0.081 | -0.376 | 0.235 |
| work and interests-insight | 0.256 | -0.128 | 0.573 |
| retardation-agitation | -0.014 | -0.339 | 0.339 |
| retardation-psychic anxiety | -0.091 | -0.391 | 0.225 |
| retardation-somatic anxiety | -0.190 | -0.499 | 0.132 |
| retardation-gastrointestinal symptoms | -0.088 | -0.430 | 0.220 |
| retardation-general somatic symptoms | -0.349 | -0.610 | -0.071 |
| retardation-genital symptoms | 0.023 | -0.325 | 0.371 |
| retardation-hypochondria | 0.099 | -0.200 | 0.420 |
| retardation-weight loss | 0.126 | -0.180 | 0.411 |
| retardation-insight | -0.064 | -0.418 | 0.270 |
| agitation-psychic anxiety | -0.231 | -0.507 | 0.080 |
| agitation-somatic anxiety | 0.003 | -0.343 | 0.335 |
| agitation-gastrointestinal symptoms | -0.102 | -0.378 | 0.189 |
| agitation-general somatic symptoms | -0.058 | -0.377 | 0.244 |
| agitation-genital symptoms | -0.206 | -0.556 | 0.102 |
| agitation-hypochondria | -0.126 | -0.411 | 0.179 |
| agitation-weight loss | 0.071 | -0.258 | 0.401 |
| agitation-insight | 0.214 | -0.077 | 0.463 |
| psychic anxiety-somatic anxiety | 0.320 | -0.010 | 0.589 |
| psychic anxiety-gastrointestinal symptoms | -0.061 | -0.364 | 0.277 |
| psychic anxiety-general somatic symptoms | 0.438 | 0.111 | 0.685 |
| psychic anxiety-genital symptoms | 0.210 | -0.094 | 0.493 |
| psychic anxiety-hypochondria | 0.340 | 0.008 | 0.602 |
| psychic anxiety-weight loss | -0.169 | -0.506 | 0.167 |
| psychic anxiety-insight | 0.053 | -0.232 | 0.346 |
| somatic anxiety-gastrointestinal symptoms | 0.144 | -0.131 | 0.426 |
| somatic anxiety-general somatic symptoms | 0.372 | 0.086 | 0.610 |
| somatic anxiety-genital symptoms | 0.076 | -0.230 | 0.368 |
| somatic anxiety-hypochondria | 0.193 | -0.139 | 0.507 |
| somatic anxiety-weight loss | -0.444 | -0.714 | -0.135 |
| somatic anxiety-insight | 0.073 | -0.261 | 0.401 |
| gastrointestinal symptoms-general somatic symptoms | 0.071 | -0.201 | 0.346 |
| gastrointestinal symptoms-genital symptoms | 0.238 | -0.085 | 0.518 |
| gastrointestinal symptoms-hypochondria | 0.168 | -0.185 | 0.468 |
| gastrointestinal symptoms-weight loss | 0.098 | -0.202 | 0.356 |
| gastrointestinal symptoms-insight | 0.149 | -0.163 | 0.455 |
| general somatic symptoms-genital symptoms | 0.146 | -0.138 | 0.427 |
| general somatic symptoms-hypochondria | 0.254 | -0.033 | 0.515 |
| general somatic symptoms-weight loss | -0.108 | -0.438 | 0.209 |
| general somatic symptoms-insight | 0.094 | -0.204 | 0.354 |
| genital symptoms-hypochondria | 0.456 | 0.128 | 0.724 |
| genital symptoms-weight loss | -0.339 | -0.540 | -0.113 |
| genital symptoms-insight | 0.097 | -0.237 | 0.403 |
| hypochondria-weight loss | -0.091 | -0.385 | 0.207 |
| hypochondria-insight | 0.210 | -0.115 | 0.499 |
| weight loss-insight | -0.034 | -0.297 | 0.278 |

**Table S4.** Average correlation stability coefficient of multiple imputation.

| Group | Metric | Mean |
| --- | --- | --- |
| With triggers | Strength | 0.369 |
|  | Closeness | 0.283 |
| Without triggers | Strength | 0.180 |
|  | Closeness | 0.141 |

**Table S5.** Frequency of the core symptoms appearing in imputation sets.

|  | With triggers | | Without triggers | |
| --- | --- | --- | --- | --- |
| Rank | Symptoms (Item No.) | f^*^ | Symptoms (Item No.) | f^*^ |
| 1 | Late insomnia (6) | 10 | Middle insomnia (5) | 6 |
| 2 | Somatic anxiety (11) | 7 | Depressed mood (1) | 4 |
| 3 | Psychic anxiety (10) | 3 | Genital symptoms (14) | 3 |
| 4 | Insight (17) | 3 | Psychic anxiety (10) | 3 |
| 5 | Early insomnia (4) | 3 | Late insomnia (6) | 3 |
| 6 | Middle insomnia (5) | 3 | Somatic anxiety (11) | 2 |
| 7 | General somatic symptoms (13) | 1 | General somatic symptoms (13) | 2 |
| 8 | - | - | Hypochondria (15) | 2 |
| 9 | - | - | Gastrointestinal symptoms (12) | 1 |
| 10 | - | - | Insight (17) | 1 |
| 11 | - | - | Guilty (2) | 1 |
| 12 | - | - | Early insomnia (4) | 1 |
| 13 | - | - | Work and interests (7) | 1 |

^*^f: Frequency.

**Table S6.** Association analysis of Cox proportional hazards model between psychosocial triggers and treatment response.

| Model | HR (95% CI) | *P* |
| --- | --- | --- |
| Model 1 | 1.156 (0.740-1.805) | 0.524 |
| Model 2 | 1.194 (0.715-1.994) | 0.499 |
| Model 3 | 1.174 (0.702-1.962) | 0.541 |

Model 1: unadjusted.

Model 2: adjusted for age, sex, alcohol category.

Model 3: adjusted for model 1 and baseline HAMD-17, baseline HAMA.

**Table S7.** Association analysis of GLMM Model between psychosocial triggers and treatment Response at different time points.

| Time | OR (95% CI)^a^ | *P* | OR (95% CI)^b^ | *P* | OR (95% CI)^c^ | *P* |
| --- | --- | --- | --- | --- | --- | --- |
| v2 | 1.40  (0.41-4.76) | 0.593 | 1.01  (0.27-3.82) | 0.984 | 1.13  (0.30-4.18) | 0.859 |
| v3 | 1.64  (0.49-5.48) | 0.425 | 1.68  (0.44-6.46) | 0.448 | 1.53  (0.41-5.75) | 0.529 |
| v4 | 1.21  (0.21-6.87) | 0.830 | 1.75  (0.28-10.80) | 0.547 | 1.58  (0.26-9.58) | 0.619 |
| v5 | 6.35  (0.32-126.96) | 0.227 | 7.91  (0.40-157.60) | 0.175 | 7.21  (0.38-136.86) | 0.188 |
| v6 | NA | - | NA | - | NA | - |
| v7 | NA | - | NA | - | NA | - |

a. Model was unadjusted. b. Model was adjusted for age, sex, alcohol category. c. Model was adjusted for age, sex, alcohol category, baseline HAMD-17 and baseline HAMA.

NA: Not applicable. Possibly caused by data sparsity.


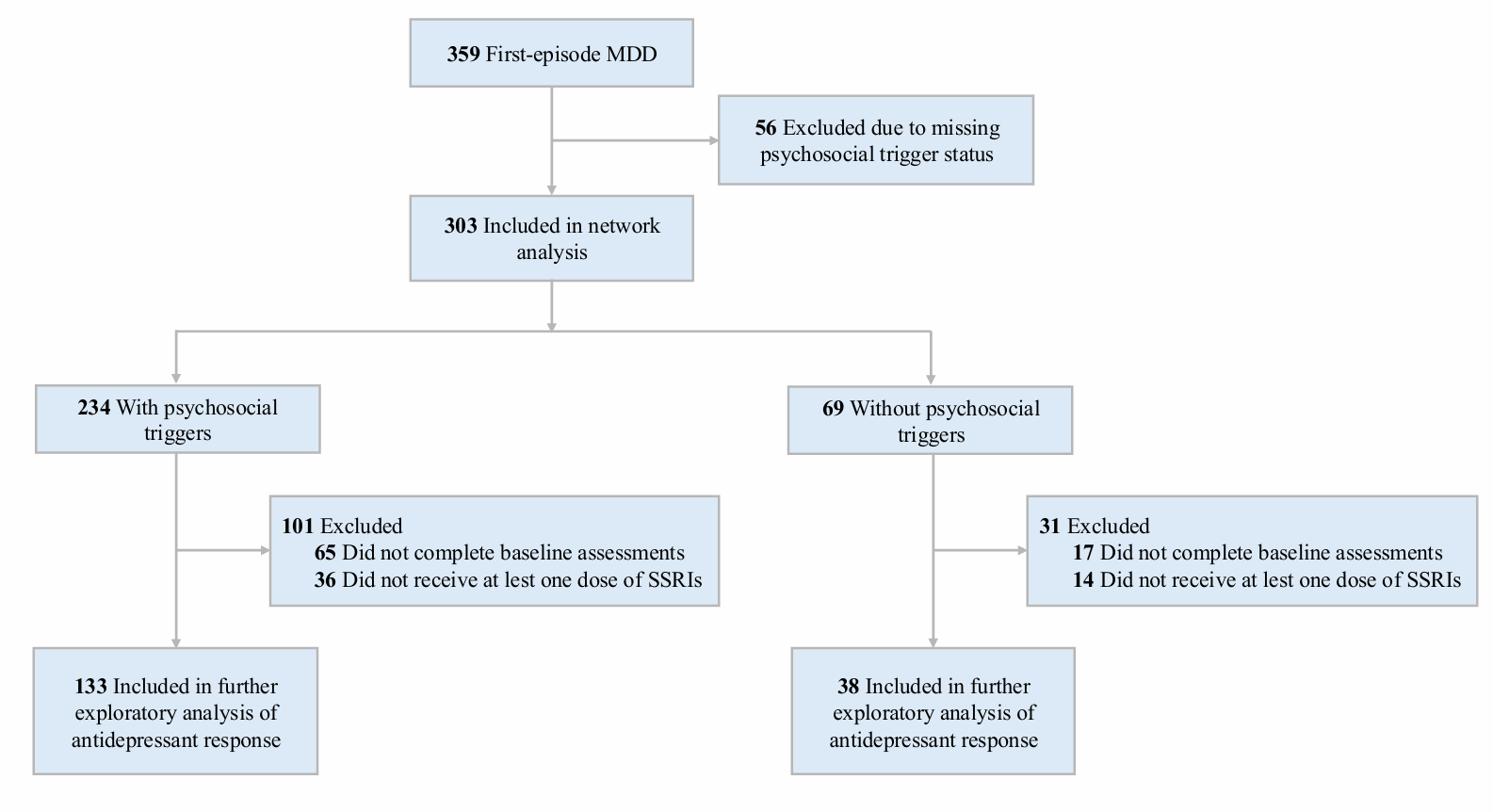


**Figure S1.** Flowchart for inclusion and exclusion of the population.
